# Molecules That Cause or Prevent Parkinson's Disease

**DOI:** 10.1371/journal.pbio.0020401

**Published:** 2004-11-16

**Authors:** Mark R Cookson

## Abstract

An overview of the molecules and associated cell biology underlying neuron death in Parkinson's Disease

The consequence of Parkinson's disease (PD) is well described: a progressive movement disorder that, whilst responding to symptomatic therapy, chronically disables its sufferers and adds an enormous economic burden in an aging society. We have some clues to the process underlying the disease from the snapshot provided by postmortem studies of diseased brains. Groups of neurons in specific brain regions are lost, notably those that produce dopamine in a part of the midbrain called the substantia nigra. Those neurons that do survive to the end of the disease course contain accumulations of proteins and lipids within their cytoplasm. Named after their discoverer, these “Lewy bodies” are one piece of evidence that protein aggregation is related to the ongoing disease process.

In contrast, the causes of PD are poorly defined except in those rare variant forms that are clearly genetic. Several families have been described where PD-like syndromes are inherited in either a dominant or recessive fashion, and four of the underlying genes have been identified. The precise relationships between these different syndromes are complex and are the subject of some controversy. For example, it is not clear whether all the genetic diseases given PARK nomenclature have Lewy bodies and should be considered “true” PD—the term parkinsonism is preferred for these syndromes (Hardy and Langston 2004). For the purposes of this primer, I will concentrate on the molecular biology of the genes linked to PD rather than disease etiology. However, my assumption is that symptoms of the disease are a reflection of neuronal dysfunction, and that in the disease state the balance between damage and survival tips in the direction of cell loss. Whilst dominant mutations overwhelm the ability of cells to survive, recessive mutations result in the absence of protective proteins and make the neuron grow weaker.

## Aggregation of α-Synuclein in Neurodegeneration

On the detrimental side of the cell survival equation is the PD gene that was discovered first, α*-synuclein*. The synaptic protein encoded by this gene, α-synuclein, is prone to aggregation, and, as is the case for other aggregating proteins, mutations in α-*synuclein* are associated with dominantly inherited disease. Related to this, α-synuclein is a major protein component of Lewy bodies. The phenotype of patients with α-*synuclein* mutations varies from PD to a more diffuse Lewy body disease in which pathology is detected in the cerebral cortex and other areas of the brain. Mutations in α-*synuclein* include three point mutations (A30P, E46K, and A53T) and multiplication of the wild-type (normal) gene. All of these mutations increase the tendency of α-synuclein to aggregate, suggesting that disease is a consequence of protein aggregation. An interesting example is the triplication of the wild-type gene: toxicity and aggregation can both be driven by increased expression and are thus qualitative, not quantitative, effects (Singleton et al. 2003). The fact that the wild-type protein can aggregate suggests that the process is fundamentally similar for both inherited and sporadic PD in which wild-type α-synuclein is also present in Lewy bodies. Several commentators have suggested that non-genetic risk factors may also promote damage via their effects on (wild-type) α-synuclein conformation or aggregation (e.g., Di Monte 2003). This reinforces the notion that α-synuclein is central to the pathogenesis of both sporadic and familial PD.

There is some controversy about the exact nature of the toxic species produced by α-synuclein, as one point mutation (A30P) behaves differently from the others. Instead of forming fibrils, which are insoluble, high-molecular-weight species, A30P forms relatively soluble, partially aggregated species (Conway et al. 2000). These intermediate-sized protein aggregates are referred to as oligomers or protofibrils. Some authors have argued that since A30P causes disease, oligomers/protofibrils are the authentic toxic species. It is generally assumed that fibrils are the form of α-synuclein deposited into Lewy bodies, but whether Lewy bodies damage cells is controversial. One possibility is that by sequestering α-synuclein into this insoluble body and compartmentalizing the potentially toxic species away from possible targets in the cytoplasm, the Lewy body represents an attempt of the cell to protect itself (Olanow et al. 2004).

Whether the Lewy body is damaging or neuroprotective, there are clearly several possible targets for toxic α-synuclein within the cell. For example, aggregated α-synuclein can permeabilize cellular membranes and thus might damage organelles (Volles and Lansbury 2003). Mitochondrial function and synaptic transmission may be especially affected, and both of these can secondarily increase oxidative stress within the cytosol (Greenamyre and Hastings 2004). When overexpressed, mutant α-synuclein can inhibit the proteasome (Petrucelli et al. 2002), a multiprotein complex that degrades many unwanted or inappropriate proteins in cells. Mutant forms of α-synuclein also inhibit chaperone-mediated autophagy, another important protein turnover pathway that involves lysosomes (Cuervo et al. 2004). Between these two effects, it is likely that cells with aggregated α-synuclein will become less able to handle damaged or misfolded proteins. It is also possible that other cellular processes that we have not yet identified are affected by the presence of this protein that has such an innate tendency to aggregate.

Presumably, neurons require α-synuclein for their normal function and thus cannot simply dispense with this protein that has toxic properties, although mice in which the α-*synuclein* gene is knocked out have no obvious deficits (see Dauer and Przedborski [2003] for discussion).

## Parkin, DJ-1, and PINK1 in Neuroprotection

Evolution has provided cells with many ways to protect themselves. As we will see, mutations that cause recessive diseases result in the loss of these neuroprotective functions. The genes involved in recessive parkinsonism are, in order of discovery, *parkin, DJ-1,* and *PINK1*. The three protein products of these genes all have different functions, thus implicating several different cellular functions in neuroprotection. Parkin is an E3 ubiquitin–protein ligase, promoting the addition of ubiquitin to target proteins prior to their degradation by the proteasome. The identification of parkin's function was facilitated by the observation that the protein contains a RING finger (Zhang et al. 2000), a common motif amongst this class of E3 enzymes. Several parkin substrates have been proposed, and at least two are damaging to neurons if they are allowed to accumulate (Dong et al. 2003; Yang et al. 2003). Therefore, our best evidence to date indicates that parkin benefits neurons by removing proteins that might otherwise damage the cell. In fact, expression of parkin is neuroprotective in a number of contexts, and there is even evidence for a beneficial effect of this E3 ligase on mitochondrial function (Shen and Cookson 2004).

Data on PINK1 are limited, but the protein contains two motifs that indicate its likely cellular role. At the amino-terminus of PINK1 is a mitochondrial-targeting sequence, and mitochondrial localization has been confirmed in the one study published to date (Valente et al. 2004). Most of the rest of PINK1 is a Serine/Threonine protein kinase domain, followed by a short carboxy-terminal region of unclear significance. The substrates of PINK1 have not yet been identified, but presumably phosphorylation of these substrates controls some critical function for neuronal survival. In their paper, Valente and colleagues show that PINK1 decreases damage to mitochondria induced by proteasome inhibition, but a recessive mutant PINK1 is unable to protect cells.

The discussion of protein functions gets more complicated in the case of DJ-1. Unlike parkin or PINK1, there are no motifs within DJ-1 that hint strongly at a single function. Instead, *DJ-1* is a member of a large superfamily of genes with several different functions across species (Bandyopadhyay and Cookson 2004). These include proteases in thermophilic bacteria, transcription factors, and chaperones that promote protein refolding. Several research groups have published data in support of DJ-1 having one or more of these activities, including the report, published in this issue of *PLoS Biology*, that DJ-1 is a molecular chaperone that regulates α-synuclein, among other molecules (Shendelman et al. 2004). It is not yet firmly established which activity of DJ-1 is most relevant to recessive parkinsonism. The important function of DJ-1 might be unrelated to any of the above activities. For example, there are several roles of this protein in modulation of transcriptional responses, which may be critical in maintaining neuronal viability (Bonifati et al. 2003 and references therein)

DJ-1 is also known to be responsive to oxidative conditions, under which cysteine residues are oxidized to form cysteine-sulfinic acids. There is some discussion about which cysteine residue is oxidized; the most likely is cysteine 106, which is present in a nucleophile elbow in the protein. We have suggested that modifying this residue precludes DJ-1 oxidation under mild conditions and also blocks the neuroprotective activity of DJ-1 against mitochondrial toxicity (Canet-Aviles et al. 2004). Therefore, whatever the function of DJ-1, it seems to be related to oxidation. In support of this idea, cells with *DJ-1* knocked out show increased sensitivity to oxidative stress (Yokota et al. 2003). Another study published in this issue of *PLoS Biology* shows that dopamine neurons differentiated from embryonic stem cells lacking functional DJ-1 are especially sensitive to oxidative stress (Martinat et al. 2004).

This discussion indicates that the genes responsible for recessive parkinsonism all have different functions but are all, in a broad sense, neuroprotective. A very difficult question to answer is whether this has anything to do with α-synuclein. We have shown that parkin can mitigate the toxicity of mutant α-synuclein (Petrucelli et al. 2002). Although there are reports that a proportion of α-synuclein is a parkin substrate (Shimura et al. 2001), most of the protein is not degraded by the ubiquitin-proteasome system. Recent evidence points, instead, to an important role of the lysosome, the other major pathway within cells for degrading unwanted proteins, in clearing α-synuclein (Cuervo et al. 2004). On balance, therefore, there is no direct evidence that parkin controls α-synuclein toxicity by an effect on protein levels within the cell. Furthermore, parkin does not just prevent α-synuclein toxicity: it is beneficial against several other stresses (discussed in Shen and Cookson 2004), leading to the possibility that this protein protects neurons against more than just the processes implicated in PD.

It has also been suggested that DJ-1 can prevent the accumulation of aggregated α-synuclein and that cysteine 53 is critical for this activity (Shendelman et al. 2004). However, DJ-1 is not just a chaperone for α-synuclein; it can also promote refolding of citrate synthase, glutathione transferase, and neurofilament light. Other research groups have reported similar findings (Olzmann et al. 2004), although there are differences between these studies in which cysteine residues are thought to be required for DJ-1 function. Given that there are some differences in these results, further clarification of the role for DJ-1 in α-synuclein-mediated toxicity is needed. More generally, we have to bear in mind that whether recessive parkinsonism has anything to do with α-synuclein is still an open question. What is clear is that some neurons rely on *parkin, DJ-1,* or *PINK1* to protect themselves against the many stresses that they face. However, mutations in these genes do not cause generalized neurodegeneration; in fact, they tend to be more restricted and less progressive than, for example, α-synuclein mutations. This suggests, at least to my mind, that recessive mutations indicate something about the neurons that are damaged in these disorders. Why is this of more than academic importance? Perhaps by identifying the proximal events that are sufficient to cause a specific set of neurons to degenerate, we might begin to design therapies that address the underlying degeneration in PD and not just the consequences.

**Figure 1:**
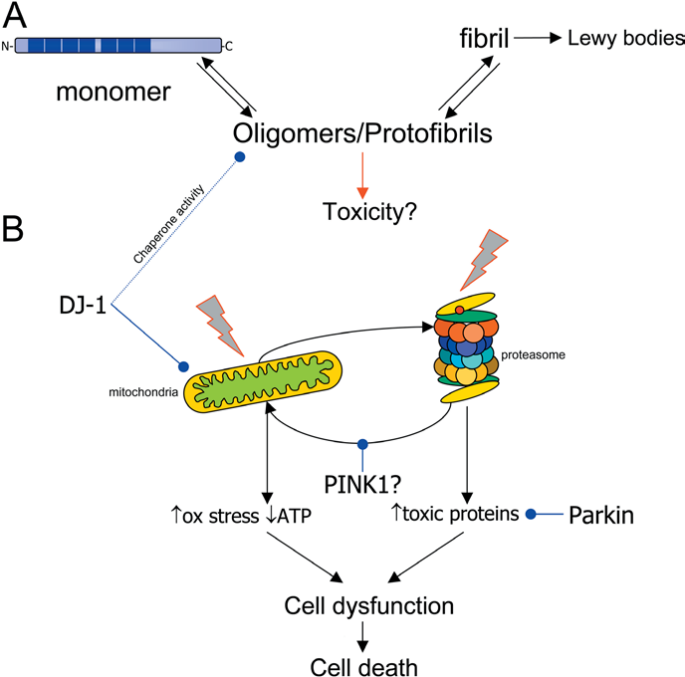
Molecules That Cause or Prevent Parkinson's Disease (A) shows a simplified, linear view of the aggregation pathway of α-synuclein (in blue). The monomer of α-synuclein is a natively unfolded protein with several repeats, shown by dark bars on the monomer. The protein has an innate tendency to aggregate with other molecules of α-synuclein, first into oligomers (also known as protofibrils), then into fibrils. It is the fibrillar forms of α-synuclein that are deposited into the classic pathological structures of PD, Lewy bodies. There are several studies that suggest that the oligomeric intermediates are the major toxic species, although this is not certain. (B) shows the recessive mutations associated with parkinsonism and their possible relationships to subcellular targets, either mitochondria (left) or the proteasome (right). Insults to either of these can cause cellular damage and may interact. For example, proteasome inhibitors can cause mitochondrial damage, which can be antagonized by PINK1. Parkin can promote the turnover of proteasomal substrates, and DJ-1 can prevent mitochondrial damage. Quite whether (B) relates to (A) is not clear, but recent results with DJ-1 imply that DJ-1 has chaperone activity towards oligomers of α-synuclein (see text). Although there is much to be done to resolve the order of these events, it is likely that, either alone or in concert, damage to multiple cellular pathways leads to neuronal dysfunction and, eventually, cell death.

## Abbreviation

PD: Parkinson's disease
